# Nightly treatment of primary insomnia with prolonged release melatonin for 6 months: a randomized placebo controlled trial on age and endogenous melatonin as predictors of efficacy and safety

**DOI:** 10.1186/1741-7015-8-51

**Published:** 2010-08-16

**Authors:** Alan G Wade, Ian Ford, Gordon Crawford, Alex McConnachie, Tali Nir, Moshe Laudon, Nava Zisapel

**Affiliations:** 1 CPS Research, Glasgow, UK; 2 Robertson Centre for Biostatistics, University of Glasgow, Glasgow, UK; 3 CPS Research, Glasgow, UK; 4 Neurim Pharmaceuticals Ltd, Tel-Aviv, Israel; 5 Department of Neurobiology Faculty of Life Sciences, Tel Aviv University, Tel-Aviv, Israel

## Abstract

**Background:**

Melatonin is extensively used in the USA in a non-regulated manner for sleep disorders. Prolonged release melatonin (PRM) is licensed in Europe and other countries for the short term treatment of primary insomnia in patients aged 55 years and over. However, a clear definition of the target patient population and well-controlled studies of long-term efficacy and safety are lacking. It is known that melatonin production declines with age. Some young insomnia patients also may have low melatonin levels. The study investigated whether older age or low melatonin excretion is a better predictor of response to PRM, whether the efficacy observed in short-term studies is sustained during continued treatment and the long term safety of such treatment.

**Methods:**

Adult outpatients (791, aged 18-80 years) with primary insomnia, were treated with placebo (2 weeks) and then randomized, double-blind to 3 weeks with PRM or placebo nightly. PRM patients continued whereas placebo completers were re-randomized 1:1 to PRM or placebo for 26 weeks with 2 weeks of single-blind placebo run-out. Main outcome measures were sleep latency derived from a sleep diary, Pittsburgh Sleep Quality Index (PSQI), Quality of Life (World Health Organzaton-5) Clinical Global Impression of Improvement (CGI-I) and adverse effects and vital signs recorded at each visit.

**Results:**

On the primary efficacy variable, sleep latency, the effects of PRM (3 weeks) in patients with low endogenous melatonin (6-sulphatoxymelatonin [6-SMT] ≤8 μg/night) regardless of age did not differ from the placebo, whereas PRM significantly reduced sleep latency compared to the placebo in elderly patients regardless of melatonin levels (-19.1 versus -1.7 min; *P *= 0.002). The effects on sleep latency and additional sleep and daytime parameters that improved with PRM were maintained or enhanced over the 6-month period with no signs of tolerance. Most adverse events were mild in severity with no clinically relevant differences between PRM and placebo for any safety outcome.

**Conclusions:**

The results demonstrate short- and long-term efficacy and safety of PRM in elderly insomnia patients. Low melatonin production regardless of age is not useful in predicting responses to melatonin therapy in insomnia. The age cut-off for response warrants further investigation.

## Background

Insomnia is a common disorder, the diagnosis of which is based on a patient's complaint of difficulty in initiating or maintaining sleep or sleep that is of inadequate quality (non-restorative sleep). The sleep disturbance should have been present for at least 1 month and be associated with a negative impact on functioning the following day. Insomnia is the most common sleep disorder in the USA. About one-third of the adult population has experienced insomnia at some time and approximately 10% have a persistent problem.

Melatonin (N-acetyl-5-methoxytryptamine) is the major hormone produced nocturnally by the pineal gland in a process driven by the biological clock residing in the suprachiasmatic nuclei (SCN). Melatonin is a sleep regulator and signal of darkness in humans [1]. Thus, the circadian rhythm in the synthesis and secretion of melatonin is closely associated with the sleep rhythm in both sighted and blind subjects [2,3]. Melatonin promotes sleep in humans [4,5], presumably by inhibiting circadian wakefulness mechanisms [6,7] and affects the activity of brain networks compatible with sleep induction [8,9]. Exogenous melatonin has clock-shifting effects and may advance or delay the sleep phase depending on the time of administration according to the Phase-Response Curve [10].

Endogenous melatonin levels decrease with age, presumably due to an age-related decline in SCN circadian rhythmic functions or the calcification of the pineal gland [11-18]. Since endogenous melatonin has beneficial effects on sleep in man and helps stabilize the physiological circadian rhythms, including the sleep-wake cycle in humans, decline in melatonin levels may contribute to the common complaint of poor sleep quality seen amongst the elderly [13,15,17]. This raised the possibility of improving sleep in elderly patients with insomnia by appropriately timed treatment with melatonin.

Early studies have shown an improvement of sleep in elderly insomnia patients by prescribing very high doses of melatonin [19]. Later studies, mostly done with lower doses of immediate release melatonin preparations and in younger people suggested a limited efficacy in primary insomnia with short-term use (4 weeks or less)[20].

Prolonged-release melatonin (PRM 2 mg), a formulation that releases melatonin gradually in the gut when administered orally in order to introduce melatonin into the circulation over the following 8-10 h, has proven efficacious and safe for the short-term treatment (3 weeks) of adults aged 55 years and older who have primary insomnia [21-25]. It has been licensed since June 2007 in Europe and in other countries for the short-term treatment of primary insomnia characterized by poor quality of sleep in patients who are aged 55 years or above. Some young patients also may have low melatonin levels and the important clinical question is whether the population most likely to respond is defined by age or by low melatonin levels. Another important issue with the use of melatonin in clinical practice, considering the chronic nature of insomnia at older age, is whether long-term use is justified and safe.

This study was therefore designed to elucidate whether PRM efficacy is related to low endogenous melatonin levels or age, which is associated with declining melatonin production. The maintenance of efficacy and the safety of PRM beyond the acute treatment were also investigated.

In healthy adults, the nocturnal melatonin production rate is normally 10 to 80 μg/night and urinary excretion of the main melatonin metabolite, 6-sulphatoxymelatonin (6-SMT) is 8 to 56 μg/night but with wide variability [11,13,16,17]. Thus, low endogenous melatonin in adults was defined as urinary excretion of the major melatonin metabolite of <8 μg 6-SMT per night.

## Methods

### Study design

This was a randomized, double-blind, parallel group clinical trial comprising a 2-week, single-blind, placebo run-in period (Baseline), a 3-week double-blind treatment period (treatment weeks 1-3) followed by a 26- week double-blind extension period (treatment weeks 4 to 29) in which patients were randomized to receive PRM (Circadin^® ^2 mg, Neurim Pharmaceuticals Ltd, Tel Aviv, Israel) or placebo, given orally as one tablet per day 2 h before bedtime, and a 2-week single-blind placebo run-out period (withdrawal). The study protocol and relevant documents were approved by Huntingdon Multi-centre Research Ethics Committee, Cambridge, UK. Participants provided written informed consent.

### Study subjects

Patients were recruited from Glasgow and the surrounding areas (West of Scotland) and were pre-screened by telephone using the Sleep History Questionnaire (SHQ) within 1 month of the baseline screening visit. The SHQ was adapted from The Management of Insomnia Guidelines for Clinical Practice [26]and resembled that recommended by Clinical Practice Guideline - Adult Insomnia [27,28]. Suitable patients were invited to Visit 1 during which they were consented and assessed for inclusion.

Men and women aged between 18 and 80 years suffering from primary insomnia according to the Diagnostic and Statistical Manual for Mental Disorders (DSM-IV) criteria with sleep latency longer than 20 min were included in the study.

The major exclusion criteria for the study included the use of benzodiazepine or non-benzodiazepine hypnotics within the previous 2 weeks or any psychoactive treatment within the previous 3 months, sleep disorders associated with a psychiatric disorder (for example, depression, anxiety, dementia), sleep disorders secondary to another medical condition (for example, sleep apnoea, circadian rhythm sleep disorder), use of prohibited concomitant medication [psychotropic treatments - neuroleptics, antiepileptics, barbiturates, antidepressants, anxiolytics and lithium, first generation antihistamines, hypnotics or treatments used as a hypnotic (for example, all benzodiazepines, zopiclone, zolpidem and zaleplon, barbiturates, buspirone and hydroxyzine)] or excessive alcohol consumption, any chronic medical condition that was likely to be the cause of the sleep problem (for example, chronic pain, benign prostatic hypertrophy) or might interfere with the conduct of the study or a lifestyle likely to interfere with sleep patterns (for example, shift work, jet-lag).

A four-step process was used for screening out patients with secondary sleep disorders including depression and other sleep disorders in the study according to DSM-IV criteria.

Step 1: The initial prescreening for primary insomnia as defined in DSM-IV was performed on a telephone interview and was based on the SHQ. The SHQ characterizes the primary sleep complaint according to the differential diagnostic criteria (DSM-IV and International Classification of Diseases-10) and also helps in differentiating primary insomnia from insomnia due to medical and psychiatric disorders (including depression and anxiety) and specific insomnia disorders such as circadian rhythm disorders, movement disorders, parasomnias and breathing related sleep disorders.

Step 2: At the screening visit, a physical examination was performed by a qualified clinician to exclude patients with physical causes of insomnia.

Step 3: At the screening visit the patients went through a detailed psychological assessment that included the Raskin Depression scale, Covi anxiety scale and the Mini Mental State (MMS) in order to exclude psychiatric disorders, including depression anxiety and dementia. In addition, a history of severe psychiatric disorders, especially psychosis, anxiety and depression were major exclusion criteria.

Step 4: Patients who were using psychotropics (neuroleptics, antiepileptics, barbiturates, antidepressants, anxiolytics or lithium) in the 3 months before the study were excluded. A urine drug screen for benzodiazepines and morphine derivatives was undertaken at baseline. Patients with a positive result were excluded. Hypnotic use was monitored throughout the study. Patients were asked at each visit whether they took a hypnotic beside medication and were withdrawn if they did. The common analgesics used in UK for self limiting intermittent problems such as headache frequently contain codeine. Patients for whom pain was a cause of insomnia were excluded from the study. However, due to the long-term nature of the study intermittent use of common analgesics was allowed.

The screening and run-in periods were used to wash-out previously administered medicinal products which were incompatible with the trial, for confirmation of a stable disease and compliance with study medication and procedures and for the qualitative and quantitative baseline assessments of patients. Patients with major short-term fluctuations of their condition and non-compliance with study procedures were excluded. A history of sleep latency of >20 min, required for patients inclusion, was assessed once in the telephone interview (SHQ), confirmed at the screening and then at the baseline visits using the PSQI [28-30]. Eligible patients entered the baseline screening run-in period and received 2 weeks of single-blind treatment with placebo. Patients still eligible after the 2-week placebo run-in and who were compliant with respect to treatment, had a negative drug screen and correctly completed study assessment forms were randomized in a 1:1 ratio to receive either PRM 2 mg or placebo for 3 weeks in a double-blind manner. Randomization was stratified by trial site, 6-SMT levels (low ≤8 μg versus high >8 μg/night) and age group (< 65 versus ≥65 years).

After the 3-week treatment period, completing patients were allowed to proceed into the extension period. All PRM patients stayed on PRM and all placebo patients were randomised in a 1:1 ratio to receive either PRM 2 mg or placebo for 26 weeks, resulting in a 3:1 ratio of PRM to placebo.

At the end of the extension period, all patients received 2 weeks of single-blind placebo in the run-out period to evaluate withdrawal effects. The overall duration of the study was 33 weeks.

Patients were instructed to take one tablet daily of study medication orally, 1-2 h before going to bed (preferably between 2100 h and 2200 h) and after food, and were asked to fill in a diary each morning, reporting on sleep latency, sleep maintenance, total sleep time, time going to bed, sleep offset time, refreshed on waking score, morning alertness score, and sleep quality in the previous night).

The treatment period was double-blind with two parallel treatment groups. Selection for a treatment group was determined by a computer generated randomization list in a 1:1 ratio (PRM mg to placebo). The list was constructed using the method of randomized permuted blocks. Randomization was stratified by trial site, 6-SMT levels (low/high) and age group (< 65/> 65). A centralized randomization system (Interactive Voice Response System [IVRS]) was used. Sites called the IVRS, using a toll-free number. The patient's status: trial site, 6-SMT level (low excretors/high excretors) and age (< 65/>65) were entered into the IVRS. The IVRS randomised the patients and provided a double-blind treatment kit assignment.

By the end of the 3 week treatment period, the PRM patients remained on the active medication and placebo patients were randomized again for the double-blind extension period. Selection for a treatment group was determined by a computer generated randomization list in a 1:1 ratio (PRM 2 mg to placebo). This procedure was performed for all participants using the centralized IVRS randomization system keeping the patient and the study personnel (investigator and nurses) blind to the allocated treatment. The list was constructed using the method of randomized permuted blocks.

Blinding was maintained by use of a matching placebo identical in appearance taste and smell to the active medication and use of an independent IVRS to allocate randomized treatment. During the 33 weeks of the study (run-in, double-blind treatment, double-blind extension and run-out), patients were blinded regarding the type of medication they were receiving (placebo or active). During the double-blind treatment and extension periods, the investigator(s) and their staff were also blinded regarding the treatment administered (double-blind). However, they were aware that the patient was receiving placebo during the run-in and run-out periods.

The blinding was not to be broken (unless in an emergency) until the database was locked to perform planned analyses. The IVRS was used to break a code in case of emergency. This was to be done only when the investigator decided that knowledge of which study treatment the patient had been randomized to, would affect the management of an adverse experience. There were three people for whom the blind was broken all due to a serious adverse event (SAE), two were on PRM and one was on a placebo.

### Endpoints

The main objective of this study was to assess the effects of short-term (3-week) therapy with PRM versus placebo on patient reported sleep latency (sleep diary) in their natural setting, in patients with low endogenous melatonin levels (≤8 versus >8 μg urinary 6-SMT/night) and in elderly patients (65-80 years old). Additional sleep and daytime parameters, safety and maintenance of PRM efficacy and safety over a 6-month period were also evaluated.

Efficacy variables were recorded at baseline and each visit. PSQI [29,30] global score, component scores, and questions 2 and 4 filled in by the investigator with the patient; sleep diary (National Sleep Foundation diary) parameters (daytime and night-time) were filled in by patient each day in the morning in the 7 days preceding each visit; World Health Organization (WHO)-5 Well-being Index (1998 version) [31]and Clinical Global Impression of Improvement (CGI) - Severity of Illness Scale (CGI-S) [32] were filled in by the investigator with the patient at screening and baseline and the improvement (CGI-I) at each subsequent visit.

The PSQI has been recommended as an essential measure for global sleep and insomnia symptoms in recent expert consensus recommendations for a standard set of research assessments in insomnia [28]. It comprises nine questions relating to the patient's usual sleep habits during the previous 2 weeks; the second and third weeks of active treatment. It addresses possible reasons for trouble in sleeping as well as daytime behaviour. An algorithm is used to calculate seven component scores and these are added to give a global PSQI score. The PSQI component scores, Question 2 (Sleep Latency) and Question 4 (Total Sleep Time) after 3 weeks' double-blind treatment, and the change from baseline levels of these parameters. It has been shown that each of the PSQI individual component scores measures a particular aspect of the overall construct. Furthermore, control subjects differ from insomnia patients in all individual components [29]. However, the correlation between individual items and global score ranged from 0.83 (subjective sleep quality) to 0.07 (cough or snore during sleep) [29]. In the evaluation of the drug effects it was therefore interesting to look at each component.

Safety variables and vital signs (pulse, blood pressure) were assessed at each visit including spontaneously reported adverse events (AEs); unusual events and AEs observed by the investigator. Physical examination was performed at screening, end of run-in, after three and 29 treatment weeks and at discontinuation. An electrocardiograph was recorded at end of run-in, after three, seven and 29 treatment weeks and at discontinuation. Vital signs (pulse, blood pressure) were recorded at all visits. Laboratory tests (haematology, biochemistry and urinalysis) were assessed at screening, after three, seven and 29 treatment weeks and at discontinuation. Endocrine evaluations were performed at screening and after 29 treatment weeks in 80 patients who were not using any hormonal contraceptives or hormonal replacement therapies and who were not suffering from any significant endocrine disease. Cortisol was assessed at screening and after 29 treatment weeks in 56 patients before and after synacthen test. The Tyrer scale [33]was completed by the investigator after 29 treatment weeks and at withdrawal.

### Statistical issues

The predefined primary efficacy variable was the comparison of sleep latency as measured by the sleep diary at 3 weeks treatment weeks with PRM (2 mg) or placebo in the pre-defined subgroups of patients who were low excretors of melatonin regardless of age (primary endpoint) and the patients aged 65-80 years, regardless of melatonin levels. The comparison was done using a linear regression model with terms for treatment (PRM versus placebo), baseline sleep latency and age group (≥65 or <65 - only for the primary endpoint). In compliance with US Food Drug Administration regulatory procedures, no correction for multiple comparisons were performed for the primary outcome measure.

All other efficacy endpoints were pre-defined as exploratory and aimed at confirming the results of the primary analysis using additional instruments (for example, PSQI) or adding information on other aspects of the sleep and daytime consequences of the treatment including: (1) time going to bed and sleep offset times, sleep maintenance, total sleep time, sleep quality and morning alertness from the sleep diaries; (2) the PSQI global score; (3) PSQI questions 2 (sleep latency in minutes) and 4 (total sleep time in minutes) and the individual PSQI components; (4) the CGI-I score assessed by the clinician at three to 29 treatment weeks) quality of life derived from the WHO-5 Well-being index covering positive mood, vitality and general interests.

Our main conclusion was based on sleep latency, the predefined primary variable. No correction was made for multiple statistical testing for the exploratory variables. Accordingly, the overall conclusions from the results are based on the accumulation of evidence for between-treatment differences which were, in many cases, correlated or complementary, rather than on isolated *P*-values.

#### Short-term period

Sleep latency as recorded in the sleep diary was summarized for low excretors aged 18-80 years and for patients aged 65-80 years, at baseline after the 2-week run-in period and after 3 weeks double-blind treatment (actual and change from baseline) for each treatment group and, as a whole, using descriptive statistics for continuous variables. At each visit, the mean value of the 7 days prior to the visit was used. Sleep latency as measured by the sleep diary after 3 weeks double-blind treatment was compared using a linear regression model with terms for treatment (PRM versus placebo), baseline sleep latency and age group (≥65 or <65 years).

The other short-term variables were summarized by the mean values at baseline and after 3 weeks of double-blind treatment (actual and change from baseline) using descriptive statistics of continuous variables for each treatment group. These included: (1) sleep diary variables, calculated as the mean of the values recorded in the 7 days prior to each study visit; (2) PSQI global, individual component, question 2 and question 4 scores; (3) WHO-5 Well-being Index score; (4) CGI-S (Visit 2) and CGI-I (Visit 3) scores.

#### Long-term period

Efficacy variables were summarized by the first randomization for outcomes at baseline and treatment weeks 4-29, or those visits at which the outcome was recorded. Summaries are given at each visit, and for changes between post-baseline visits and baseline. For those outcomes recorded at withdrawal, summaries are given for the change between treatment week 29 and withdrawal weeks. In addition, the changes of PSQI and WHO-5 between treatment week 29 and withdrawal weeks in the run-out period are summarized.

For efficacy outcomes measured at treatment weeks 4 to 29, a linear mixed effects model for repeated measures [MMRM] was used to compare outcomes at treatment weeks 4 to 29, in relation to the treatment currently received. For treatment week 3 measures, treatment was defined by the first randomization; for subsequent visits, treatment was defined by the second randomization. Each model included a random individual effect and assumed a general covariance structure for the residuals over time. For each outcome, a model was fitted which included terms for treatment, visit (as a categorical variable), the baseline values of the outcome measure, age group (≥65 or <65 years, except for analyses for the ≥65 years) and baseline 6-SMT (≤8 or >8 μg/night, except for analyses of low excretors). This model was used to estimate the global treatment effect, with a 95% confidence interval (CI) and *P*-value.

For each outcome, the above model was extended by including treatment-by-visit interaction terms. These models were used to estimate the treatment effect at each visit, with 95% CIs and *P*-values. A *P*-value for the treatment-by-visit interaction is provided, based on a likelihood ratio test. The global and visit-specific treatment effect estimates and 95% CIs are provided graphically, along with the estimated mean values and 95% CIs at each visit for each treatment group. In addition, a model was also fitted for each outcome, including a treatment-by-visit interaction, assuming a linear trend in the treatment effect over Visits 3 to 7, with the treatment effect changing by a fixed amount between each consecutive pair of visits; a likelihood ratio test *P*-value is given for this trend.

#### Safety outcomes

Adverse event data, clinical laboratory data, including hormones, vital signs and withdrawal symptoms, were summarized for all randomized study participants who took at least one dose of study medication, regardless of their subsequent participation in the study. No formal statistical testing was performed on the safety data.

#### Sample size

In order to achieve 95% power at the 5% level for the primary objective of assessing the change in sleep latency in the Intention to Treat (ITT) low excretors at Week 3, assuming a treatment effect of 19 min and a residual standard deviation of 40.6 min, 120 participants were required per treatment group. Assuming equal numbers of low and high excretors, 480 patients were required to complete treatment Week 3. Assuming a 10% dropout rate between baseline and Week 3, 540 patients would have to be randomized at baseline. In order to achieve 90% power at the 5% level for the first secondary objective of assessing the change in sleep latency in the ITT dataset of patients aged 65-80 years, assuming a treatment effect of 14 min and a residual standard deviation of 40.7 min, 179 patients ≥65 years were required per group (active and placebo). Therefore, 400 patients in this age range would need to be randomized at baseline. Assuming 45% of 540 patients already randomized would be ≥65 years old (245), an additional 150 patients would need to be randomized at baseline in this age group.

## Results

### Patient disposition and demographics

**Figure 1:**
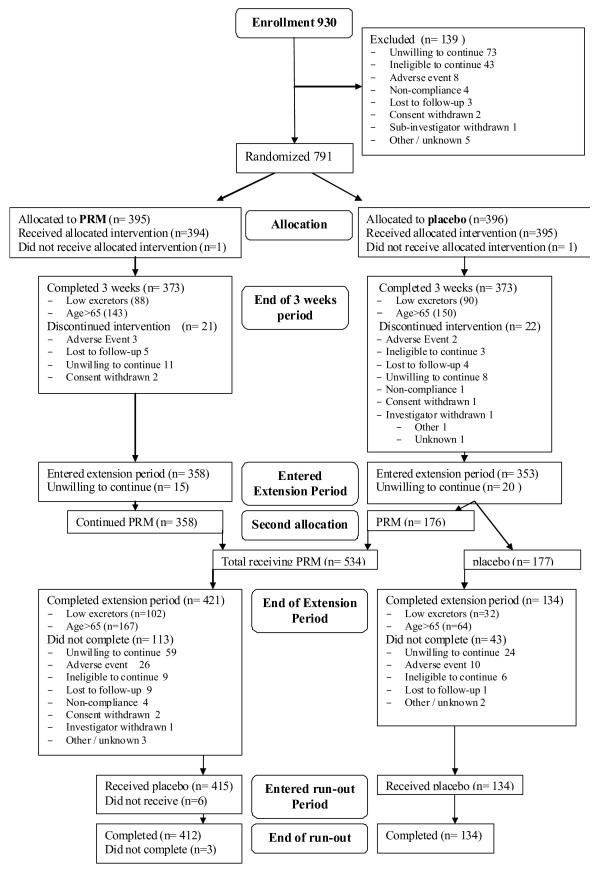
**Overall study patient disposition (CONSORT)**.

A total of 930 patients were enrolled into the study between October 2006 and December 2008 and entered the run-in period; 139 of these patients discontinued during the run-in period. The overall disposition of the patients in this study is summarized in Figure 1.

Of 791 patients in the short-term period, two were not treated with study drug and were excluded from the safety population. Of the 789 patients in the safety population, 43 (5%) were discontinued before the end of the 3-week treatment period. The most common reasons for discontinuation were withdrawal of consent (19, 44%), lost to follow-up (9, 21%) and discontinuation due to an AE (5, 12%). No other reason accounted for more than 10% of discontinuations. The pattern of discontinuation was similar for both treatment groups in baseline 6-SMT, age and gender (Figure 1). The Full Analysis Set (FAS) comprised 746 patients: 373 in the PRM group, and 373 in the placebo group. Baseline demography of the low excretors and 65-80 year-old patients are depicted in Tables 1 and 2. Patient baseline characteristics were similar between the two treatment cohorts.

**Table 1:** Baseline characteristics of the low excretor population, by treatment outcome(s): age, sex, race, height, weight, BMI and medication use.

			All	First randomization		Second randomization	
					
				PRM	Placebo	PRM	Placebo
		***N***	**172**	**86**	**86**	**127**	**39**

Characteristic	Visit						

Age (years)	1	Mean (SD)	63.8 (9.3)	63.4 (9.8)	64.2 (8.8)	63.4 (9.6)	64.3 (8.3)

Sex		*N *(%) female	128 (74.4%)	63 (73.3%)	65 (75.6%)	96 (75.6%)	28 (71.8%)

Race*		*N *(%) white	170 (98.8%)	84 (97.7%)	86 (100.0%)	125 (98.4%)	39 (100.0%)

Height (m)	1	Mean (SD)	1.63 (0.08)	1.64 (0.09)	1.63 (0.08)	1.64 (0.09)	1.62 (0.08)

Weight (kg)	1	Mean (SD)	71.4 (13.1)	71.0 (14.0)	71.8 (12.2)	70.9 (13.4)	72.8 (12.3)

BMI (kg/m^2^)	1	Mean (SD)	26.7 (4.1)	26.4 (3.9)	27.1 (4.3)	26.4 (4.0)	27.7 (4.5)

Taking any medications	1	*N *(%)	17 (9.9%)	6 (7.0%)	11 (12.8%)	10 (7.9%)	6 (15.4%)

Taking codeine†	1	*N *(%)	29 (16.9%)	13 (15.1%)	16 (18.6%)	21 (16.5%)	8 (20.5%)

Taking codeine	2	*N *(%)	26 (15.1%)	16 (18.6%)	10 (11.6%)	21 (16.5%)	5 (12.8%)

Confirmed codeine analgesic	2	*N *(%)	15 (8.7%)	8 (9.3%)	7 (8.1%)	11 (8.7%)	4 (10.3%)

*One patient missing observation.

†Two patients missing observations at Visit 1.

PRM, prolonged release melatonin; SD, standard deviation

**Table 2:** Baseline characteristics of those aged 65-80, by treatment: age, sex, race, height, weight, BMI and medication use.

			All	First randomization		Second randomization	
					
				PRM	Placebo	PRM	Placebo
		***N***	**281**	**137**	**144**	**198**	**75**

Characteristic	Visit						

Age (years)	1	Mean (SD)	71.0 (4.1)	71.1 (3.8)	70.9 (4.4)	70.9 (3.9)	70.9 (4.4)

Sex		*N *(%) female	182 (64.8%)	89 (65.0%)	93 (64.6%)	128 (64.6%)	50 (66.7%)

Race*		*N *(%) white	280 (100.0%)	137 (100.0%)	143 (100.0%)	198 (100.0%)	74 (100.0%)

Height (m)	1	Mean (SD)	1.65 (0.09)	1.65 (0.09)	1.64 (0.09)	1.65 (0.09)	1.64 (0.09)

Weight (kg)	1	Mean (SD)	73.3 (13.0)	73.1 (14.1)	73.6 (11.8)	73.2 (13.2)	74.1 (12.6)

BMI (kg/m^2^)	1	Mean (SD)	27.0 (3.8)	26.8 (3.6)	27.3 (3.9)	26.8 (3.6)	27.7 (4.2)

Taking any medications	1	*N *(%)	265 (94.3%)	130 (94.9%)	135 (93.8%)	187 (94.4%)	70 (93.3%)

Taking codeine†	1	*N *(%)	73 (26.2%)	35 (25.7%)	38 (26.6%)	50 (25.5%)	20 (26.7%)

Taking codeine	2	*N *(%)	58 (20.6%)	28 (20.4%)	30 (20.8%)	40 (20.2%)	16 (21.3%)

Confirmed codeine analgesic	2	*N *(%)	43 (15.3%)	18 (13.1%)	25 (17.4%)	25 (12.6%)	16 (21.3%)

*One patient missed observation

†Two patients missed observations at Visit 1

PRM, prolonged release melatonin; SD, standard deviation

The number of patients in the low excretors (86 per treatment group) and 65-80 year-old subgroups (PRM 137; placebo 144) of the FAS was lower than planned. The study failed to meet the design requirements for a 95% statistical power on the primary endpoint (120 low excretors per treatment group) and 90% power on the first secondary endpoint (179 patients ≥65 years per treatment group) involving sleep latency. The power reached with these sample sizes was 86% for the patients with low endogenous melatonin and 82% for the patients aged 65 and older. The 722 patients (225, 31% men; 497 women, 69%) in the FAS had a mean age of 62 years (range 20 to 80 years).

The treatment groups in the FAS were very similar regarding gender, age, race, BMI, pulse, blood pressure, ECG, medication use, medical history, and physical examination abnormalities (Tables 1 and 2). Caffeine, alcohol intake and smoking status were also generally similar between the treatment groups. The treatment groups were generally well balanced regarding demographic characteristics, pulse, ECG, blood pressure, compliance, medication use, medical history and physical examination characteristics. In this study 15.3% of the age 65-80 population and 8.7% of the low excretor population were confirmed to be taking common analgesia medications that included codeine at anytime during the study. As can be seen in the demography (Tables 1 and 2), the patients reporting usage of codeine containing medications were evenly randomized to PRM and Placebo in both randomization periods.

### Efficacy

#### Primary efficacy variable

**Figure 2:**
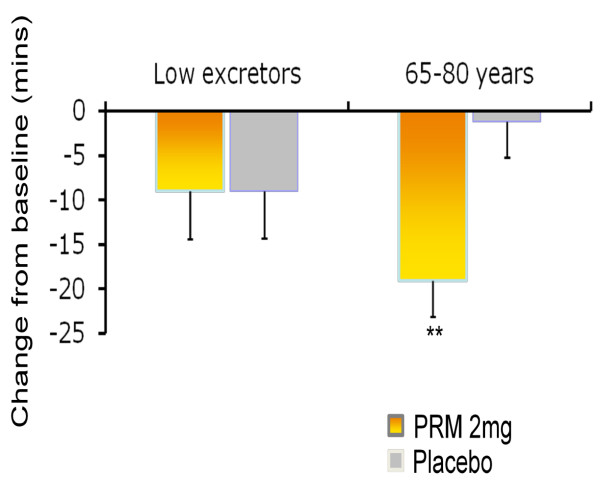
**The effect of 3 weeks treatment with prolonged release melatonin (PRM) and placebo on sleep latency in the low excretors and age 65-80 populations**. Mean+standard error of mean values of the change from baseline in sleep latency from the sleep diary following 3 weeks of double blind treatment of low excretors (*N *= 86 per group) and age 65-80 years (*N *= 137 PRM, 144 placebo) populations with PRM and placebo. Asterisks denote significant difference between PRM and placebo groups (**P < 0.01).

The effects of 3 weeks treatment with PRM or placebo on patient reported sleep latency (sleep diary) for low excretors and elderly patients are shown in Table 3. Sleep latency after 3 weeks of treatment was not significant in low excretors aged 18-80 between the PRM and placebo groups, as measured by the sleep diary, whereas a significant difference in favour of PRM was found for patients aged 65-80 years (-15.6 min; 95% CI -25.3 to -6.0, *P *= 0.002; Table 3; Figure 2).

**Table 3:** Effects of 3 weeks treatment with prolonged release melatonin (PRM) and placebo on sleep diary-recorded sleep latency in the low excretors and the elderly patients.

	Treatment		Treatment effect difference PRM - placebo; (95% confidence interval)	*P *value* effect PRM versus placebo
			
	PRM	Placebo		
**Low excretor population**				

*N*	86	86		

Baseline: mean (SD)	74.1 (54.9)	75.5 (58.5)		

Treatment: mean (SD)	65.1 (59.9)	66.5 (51.6)		

Change from baseline: mean (SD)	-9.0 (50.5)	-9.0 (48.7)	-0.6 (-14.0, 12.7)	0.924

**65-80 year population**				

*N*	137	144		

Baseline: mean (SD)	76.7 (63.7)	72.5 (51.4)		

Treatment: mean (SD)	57.6 (51.8)	70.9 (54.0)		

Change from baseline: mean (SD)	-19.1 (47.3)	-1.7 (47.8)	-15.6 (-25.3, -6.0),	0.002

*Linear regression model with terms for treatment (PRM versus placebo), baseline sleep latency and age group (≥65 or <65 - only for the low excretors).

PRM, prolonged release melatonin; SD, standard deviation.

### Other variables in the short-term period

The results of the diary, PSQI variables, CGI-I and WHO-5 Index for low excretors aged 18-80 are depicted in Tables 4 and 5. In the short-term period, low excretors aged 18-80 years did not differ significantly in the majority of variables but did have significantly fewer sleep disturbances [PSQI component 5; -0.10 (-0.18, -0.03) *P *= 0.008] and a significantly improved quality of life [WHO-5 Index score; 1.21 (0.22, 2.20), *P *= 0.016] with PRM compared to placebo (Tables 4 and 5).

**Table 4:** Sleep diary parameters in the low excretors.

				Treatment effects	
				
		Change from baseline Mean (SD)		Short term	Long term *
				
		Visit 3	Visit 7	Estimate (95% CI) *P*-value	Estimate (95% CI) *P*-value
*N*_MAX_	PRM	86	99		
	Placebo	86	31		

Sleep latency	PRM	-9.0 (50.5)	-23.6 (42.1)	-0.6 (-14.0, 12.7)	-6.7 (-16.4, 3.0)
(min)	Placebo	-9.0 (48.7)	-19.4 (79.5)	*P *= 0.924	*P *= 0.174

Sleep	PRM	-0.27 (0.77)	-0.31 (1.08)	-0.16 (-0.39, 0.08)	-0.04 (-0.24, 0.16)
maintenance	Placebo	-0.12 (1.04)	-0.34 (0.71)	*P *= 0.185	*P *= 0.677

Total sleep	PRM	0.34 (0.88)	0.70 (1.00)	9.1 (-6.1, 24.4)	13.1 (1.0, 25.2)
time (h)	Placebo	0.20 (0.91)	0.47 (1.18)	*P *= 0.236	*P *= 0.035

Sleep onset	PRM	-0.15 (0.89)	-0.46 (0.79)	-0.08 (-0.33, 0.17)	-0.16 (-0.34, 0.03)
(h)	Placebo	-0.05 (0.80)	-0.24 (1.11)	*P *= 0.530	*P *= 0.096

Sleep offset	PRM	0.09 (0.79)	0.08 (0.84)	0.04 (-0.18, 0.25)	0.07 (-0.09, 0.22)
(h)	Placebo	0.10 (0.75)	0.20 (1.00)	*P *= 0.744	*P *= 0.392

Refreshed on	PRM	-0.09 (0.46)	-0.23 (0.43)	-0.01 (-0.13, 0.11)	-0.03 (-0.12, 0.06)
waking	Placebo	-0.09 (0.40)	-0.29 (0.47)	*P *= 0.830	*P *= 0.540

Morning	PRM	-0.14 (0.67)	-0.42 (0.65)	0.01 (-0.18, 0.19)	-0.06 (-0.19, 0.08)
alertness	Placebo	-0.19 (0.63)	-0.35 (0.73)	*P *= 0.936	*P *= 0.426

Sleep quality	PRM	-0.20 (0.67)	-0.43 (0.70)	-0.04 (-0.24, 0.16)	-0.08 (-0.23, 0.06)
	Placebo	-0.16 (0.70)	-0.34 (0.60)	*P *= 0.688	*P *= 0.266

*The global treatment effect is estimated using a mixed-effect model for repeated measures and takes into account the treatment effect difference over the 26-weeks (at Visits 3, 4, 5, 6 and 7) sleep latency (diary data) are repeated for completeness

C, confidence interval; SD, standard deviation; PRM, prolonged release melatonin.

**Table 5:** Pittsburgh Sleep Quality Index (PSQI) measures, Clinical Global Impression of Improvement (CGI-I) and World Health Organization-5 Index in the low excretors.

		Change from baseline Mean (SD)		Treatment effects	
				**Short term**	Long term *
				
		**Visit 3**	**Visit 7**	**Estimate (95% CI)** ***P*-value**	**Estimate (95% CI)** ***P*-value**

N_MAX_	PRM	86	101		
	Placebo	86	31		

PSQI	PRM	-2.13 (2.89)	-3.78 (3.65)	-0.40 (-1.19, 0.38)	-0.66 (-1.30, -0.01)
Global score	Placebo	-1.62 (2.59)	-2.94 (2.91)	*P *= 0.313	*P *= 0.046

PSQI	PRM	-0.30 (0.83)	-0.72 (0.80)	-0.02 (-0.22, 0.19)	-0.13 (-0.29, 0.02)
Component 1	Placebo	-0.26 (0.67)	-0.42 (1.06)	*P *= 0.884	*P *= 0.086

PSQI	PRM	-0.37 (0.72)	-0.90 (1.02)	-0.12 (-0.33, 0.10)	-0.17 (-0.36, 0.02)
Component 2	Placebo	-0.26 (0.71)	-0.68 (0.79)	*P *= 0.278	*P *= 0.080

PSQI	PRM	-0.50 (0.89)	-0.87 (1.04)	-0.04 (-0.29, 0.21)	-0.13 (-0.33, 0.06)
Component 3	Placebo	-0.44 (0.83)	-0.65 (0.88)	*P *= 0.735	*P *= 0.169

PSQI	PRM	-0.40 (1.09)	-0.89 (1.25)	0.09 (-0.20, 0.39)	-0.01 (-0.24, 0.21)
Component 4	Placebo	-0.45 (0.99)	-0.77 (1.12)	*P *= 0.537	*P *= 0.918

PSQI	PRM	-0.09 (0.33)	-0.01 (0.48)	-0.10 (-0.18, -0.03)	-0.01 (-0.06, 0.05)
Component 5	Placebo	0.03 (0.32)	-0.03 (0.31)	*P *= 0.008	*P *= 0.811

PSQI	PRM	0.00 (0.00)	0.00 (0.00)	^†^	^†^
Component 6	Placebo	0.00 (0.00)	0.00 (0.00)		

PSQI	PRM	-0.47 (0.82)	-0.39 (0.99)	-0.17 (-0.35, 0.01)	-0.06 (-0.17, 0.05)
Component 7	Placebo	-0.24 (0.87)	-0.39 (0.84)	*P *= 0.067	*P *= 0.283

PSQI	PRM	-18.3 (52.4)	-41.3 (59.0)	-0.2 (-13.2, 12.8)	-11.6 (-22.0, -1.1)
Question 2	Placebo	-18.5 (51.7)	-33.1 (92.2)	*P *= 0.980	*P *= 0.030

PSQI	PRM	0.63 (1.10)	1.11 (1.33)	0.09 (-0.20, 0.38)	0.17 (-0.07, 0.41)
Question 4	Placebo	0.51 (0.94)	0.81 (1.08)	*P *= 0.539	*P *= 0.164

CGI-I^‡^	PRM	3.22 (1.05)	2.50 (1.19)	-0.15 (-0.46, 0.16)	-0.25 (-0.49, -0.01)
	Placebo	3.31 (0.98)	3.06 (1.21)	*P *= 0.339	*P *= 0.042

WHO-5	PRM	1.27 (3.53)	1.79 (4.27)	1.21 (0.22, 2.20),	0.91 (0.16, 1.66)
Index	Placebo	-0.03 (3.45)	1.06 (3.56)	*P *= 0.016	*P *= 0.017

Mean (standard deviation; SD) changes from baseline at Visits 3 and 7, with estimates of short-term treatment effect (linear regression model, adjusted for baseline value and age) and long-term treatment effect (mixed effects regression model, adjusted for baseline value, age and visit; global treatment effect reported).

*Global treatment effect is estimated using a mixed-effect model for repeated measures and takes into account the treatment effect difference over the 26-weeks (at Visits 3, 4, 5, 6 and 7).

^†^Regression models for PSQI Component 6 not fitted due to lack of variability in the data.

^‡^Data shown are for values recorded at each visit, and regression models do not include baseline adjustment, since CGI-I at baseline not defined.

CI, confidence interval.

The results of the diary, PSQI variables, CGI-I and WHO-5 Index for patients aged 65-80 years are presented in Tables 6 and 7. In patients aged 65-80 years, in the short-term period the PRM group showed significant advantages in sleep latency assessed by PSQI question 2 [-13.7 (-23.5, -3.9), *P *= 0.006], sleep maintenance assessed by the sleep diary [-0.17 (-0.33, 0.00), *P *= 0.046], time going to bed (hours relative to midnight) assessed by the sleep diary [-0.22 (-0.39, -0.05), *P *= 0.012], and quality of sleep as assessed by Global PSQI scores [-0.64 (-1.25, -0.02), *P *= 0.042; Tables 6 and 7].

**Table 6:** Sleep Diary parameters in the 65-80 age group.

				Treatment effects	
				
		Change from baseline Mean (SD)		Short term	Long term *
				
		Visit 3	Visit 7	Estimate (95% CI) *P*-value	Estimate (95% CI) *P*-value
N_MAX_	PRM	137	159		
	Placebo	144	61		

Sleep latency	PRM	-19.1 (47.3)	-25.9 (46.4)	-15.6 (-25.3, -6.0)	-14.5 (-21.4, -7.7)
(min)	Placebo	-1.7 (47.8)	-8.3 (61.5)	*P *= 0.002	P < 0.001

Sleep	PRM	-0.24 (0.80)	-0.31 (0.94)	-0.17 (-0.33, 0.00)	-0.09 (-0.22, 0.03)
maintenance	Placebo	-0.09 (0.78)	-0.20 (0.70)	*P *= 0.046	*P *= 0.148

Total sleep	PRM	0.34 (0.75)	0.64 (0.99)	7.0 (-3.4, 17.4)	7.5 (-0.7, 15.7)
time (h)	Placebo	0.20 (0.79)	0.41 (1.06)	*P *= 0.186	*P *= 0.073

Sleep onset	PRM	-0.22 (0.80)	-0.41 (0.75)	-0.22 (-0.39, -0.05)	-0.21 (-0.33, -0.08)
(hours)	Placebo	0.00 (0.71)	-0.12 (1.06)	*P *= 0.012	*P *= 0.002

Sleep offset	PRM	0.03 (0.81)	0.03 (0.84)	-0.16 (-0.33, 0.02)	-0.12 (-0.24, 0.00)
(h)	Placebo	0.19 (0.79)	0.21 (0.91)	*P *= 0.076	*P *= 0.051

Refreshed on	PRM	-0.10 (0.36)	-0.22 (0.42)	0.00 (-0.08, 0.08)	-0.06 (-0.12, 0.00)
waking	Placebo	-0.09 (0.37)	-0.11 (0.42)	*P *= 0.994	*P *= 0.053

Morning	PRM	-0.18 (0.52)	-0.36 (0.69)	-0.04 (-0.16, 0.07)	-0.10 (-0.19, -0.01)
alertness	Placebo	-0.11 (0.51)	-0.09 (0.60)	*P *= 0.453	*P *= 0.032

	PRM	-0.20 (0.56)	-0.39 (0.71)	-0.06 (-0.19, 0.07)	-0.08 (-0.18, 0.01)
Sleep quality	Placebo	-0.12 (0.57)	-0.17 (0.54)	*P *= 0.356	*P *= 0.082

Mean (SD) changes from baseline at Visits 3 and 7, with estimates of short-term treatment effect (linear regression model, adjusted for baseline value and age) and long-term treatment effect (mixed effects regression model, adjusted for baseline value, age and visit; global treatment effect reported)

Mean (standard deviation; SD) changes from baseline at Visits 3 and 7, with estimates of short-term treatment effect (linear regression model, adjusted for baseline value and age) and long-term treatment effect (mixed effects regression model, adjusted for baseline value, age and visit; global treatment effect reported)

*Global treatment effect is estimated using a mixed-effect model for repeated measures and takes into account the treatment effect difference over the 26-weeks (at Visits 3, 4, 5, 6 and 7) sleep latency (diary data) are repeated for completeness.

CI, confidence interval

**Table 7:** Pittsburgh Sleep Quality Index (PSQI) measures, Clinical Global Impression of Improvement (CGI-I) and World Health Organization-5 Index in the 65-80 age group.

		Change from baseline Mean (SD)		Treatment effects	
				**Short term**	Long term *
				
		**Visit 3**	**Visit 7**	**Estimate (95% CI)** ***P*-value**	**Estimate (95% CI)** ***P*-value**

*N*_MAX_	PRM	136	164		
	Placebo	144	62		

PSQI	PRM	-1.86 (2.93)	-3.34 (3.37)	-0.64 (-1.25, -0.02)	-0.70 (-1.17, -0.23)
Global score	Placebo	-1.19 (2.53)	-2.08 (2.92)	*P *= 0.042	*P *= 0.003

PSQI	PRM	-0.32 (0.71)	-0.59 (0.82)	-0.09 (-0.23, 0.05)	-0.15 (-0.25, -0.04)
Component 1	Placebo	-0.19 (0.65)	-0.34 (0.85)	*P *= 0.217	*P *= 0.006

PSQI	PRM	-0.43 (0.87)	-0.75 (0.99)	-0.23 (-0.41, -0.04)	-0.24 (-0.38, -0.10)
Component 2	Placebo	-0.22 (0.74)	-0.52 (0.95)	*P *= 0.018	*P *= 0.001

PSQI	PRM	-0.48 (0.89)	-0.86 (1.08)	-0.10 (-0.29, 0.10)	-0.10 (-0.25, 0.05)
Component 3	Placebo	-0.40 (0.89)	-0.65 (0.96)	*P *= 0.328	*P *= 0.177

PSQI	PRM	-0.32 (0.96)	-0.79 (1.22)	-0.05 (-0.27, 0.17)	-0.10 (-0.26, 0.06)
Component 4	Placebo	-0.29 (1.04)	-0.47 (1.05)	*P *= 0.638	*P *= 0.236

PSQI	PRM	0.01 (0.41)	-0.04 (0.43)	-0.05 (-0.13, 0.02)	0.00 (-0.05, 0.05)
Component 5	Placebo	0.03 (0.39)	-0.02 (0.42)	*P *= 0.162	*P *= 0.973

PSQI	PRM	-0.01 (0.12)	0.01 (0.18)	^†^	^†^
Component 6	Placebo	0.00 (0.00)	0.00 (0.00)		

PSQI	PRM	-0.30 (0.95)	-0.31 (0.94)	-0.04 (-0.19, 0.11)	-0.07 (-0.17, 0.02)
Component 7	Placebo	-0.12 (0.69)	-0.10 (0.86)	*P *= 0.636	*P *= 0.137

PSQI	PRM	-25.4 (50.9)	-32.7 (49.3)	-13.7 (-23.5, -3.9)	-12.1 (-19.1, -5.1)
Question 2	Placebo	-8.9 (48.0)	-19.0 (65.8)	*P *= 0.006	*P *= 0.001

PSQI	PRM	0.58 (1.03)	1.05 (1.29)	0.10 (-0.13, 0.33)	0.14 (-0.04, 0.32)
Question 4	Placebo	0.48 (1.00)	0.71 (1.08)	*P *= 0.381	*P *= 0.120

	PRM	3.34 (1.17)	2.70 (1.17)	-0.12 (-0.37, 0.14)	-0.20 (-0.38, -0.02)
CGI-I^‡^	Placebo	3.54 (0.85)	3.19 (1.11)	*P *= 0.364	*P *= 0.027

WHO-5	PRM	1.02 (3.73)	1.51 (4.05)	0.42 (-0.34, 1.18),	0.55 (-0.02, 1.13)
Index	Placebo	0.27 (3.15)	0.35 (4.21)	*P *= 0.281	*P *= 0.058

Mean (standard deviation; SD) changes from baseline at Visits 3 and 7, with estimates of short-term treatment effect (linear regression model, adjusted for baseline value and age) and long-term treatment effect (mixed effects regression model, adjusted for baseline value, age and visit; global treatment effect reported)

*Global treatment effect is estimated using a mixed-effect model for repeated measures and takes into account the treatment effect difference over the 26-weeks (at Visits 3, 4, 5, 6 and 7).

^†^Regression models for PSQI Component 6 not fitted due to lack of variability in the data.

^‡ ^Data shown are for values recorded at each visit, and regression models do not include baseline adjustment, since CGI-I at baseline not defined.

#### Long-term period

The results of the long-term analyses using MMRM for low excretors aged 18-80 are presented in Tables 4 and 5.

In the low excretors (regardless of age) a longer total sleep time was seen with PRM compared to placebo treated patients (estimated difference 13.1 min, 95% CI 1.0 to 25.2, *P *= 0.035; Table 4). PSQI global scores were lower (improved) in the PRM group across study visits with significant global treatment effects [-0.66 (-1.30, -0.01), *P *= 0.046; Table 5]. WHO-5 Index scores were significantly improved in PRM patients for the low excretors [0.91 (0.16, 1.66), *P *= 0.017; Table 5]. CGI-I scores were significantly lower (improved) across study visits in the PRM group compared with placebo for these patients [-0.25 (-0.49, -0.01), *p *= 0.042; Table 5]. Concerning sleep latency, there was a significant improvement with PRM over placebo in the low excretors when measured with the PSQI question 2 [-11.6 (-22.0, -1.1) min, *P *= 0.030] that was less consistently observed with the diary [-6.7 (-16.4, 3.0) min, *P *= 0.174; Tables 4 and 5].

**Figure 3:**
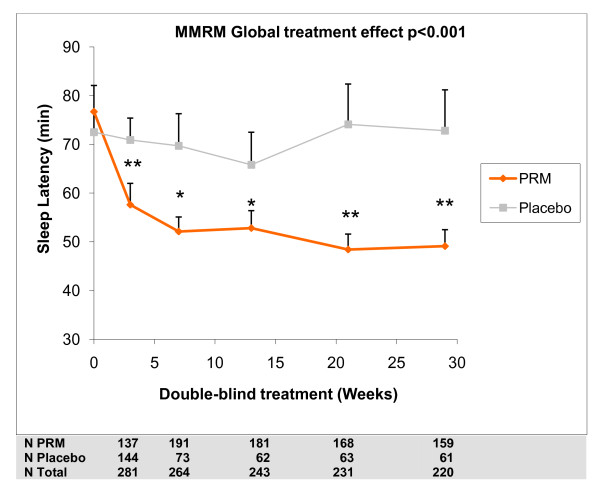
**Sleep latency during the treatment period**. Mixed Effect Model for Repeated Measures (MMRM) predicted mean values (mean + standard error of mean) for sleep latency from the sleep diary, at baseline and weeks 3-29 of the double blind treatment periods, in those aged 65-80 years. Asterisks denote significant difference between prolonged release melatonin and placebo groups (*P < 0.05 ** *P *< 0.01). Numbers of patients analysed in each treatment time point are depicted

**Figure 4:**
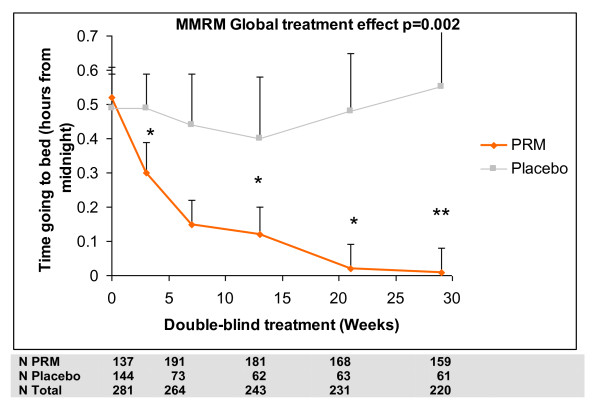
**Time of going to bed during the treatment period**. MMRM-predicted mean values (mean + standard error of mean) for time going to bed (hours relative to midnight) from the sleep diary at baseline and weeks 3-29 of the double blind treatment periods, Intention to Treat (ITT) 65-80 population. Asterisks denote significant difference between prolonged release melatonin and placebo groups (*P < 0.05 ** *P *< 0.01). Numbers of patients analysed in each treatment time point are depicted.

The results of the long-term analyses using MMRM for low excretors aged 18-80 and for patients aged 65-80 years are presented in Tables 6 and 7. In patients aged 65-80 (regardless of melatonin excretion), sleep latency throughout the long term period was significantly shorter in the PRM group as assessed by the sleep diary with a mean difference from placebo of -14.5 min (-21.4, -7.7; *P *< 0.001; Table 6). Similar results were observed with sleep latency recorded by PSQI component 2 and PSQI question 2 (Table 7) The estimated treatment effect differences for sleep latency during the extension period for these patients show incremental difference between PRM and placebo with time of treatment up to 3 months reaching plateau levels that are maintained to the rest of the 6 months period (Figure 3). A similar pattern was seen for time going to bed, with PRM patients going to bed significantly earlier than placebo patients (treatment difference -0.21 h over placebo; 95% CI -0.33 to -0.08, *P *= 0.002; Table 6 and Figure 4). There was some indication that PRM patients also woke up somewhat earlier (treatment difference -0.12 h; 95% CI -0.24 to 0.00, *P *= 0.051).

PSQI global scores were lower (improved) in the PRM group across study visits with significant global treatment effects [-0.70 (-1.17, -0.23) *P *= 0.003; Table 7].

Consistent with the improvements in quality of sleep (PSQI global score) and sleep latency (diary), components 1 (sleep quality) and 2 (sleep latency) of the PSQI were significantly lower (improved) in the PRM group across study visits in patients aged 65-80 years [-0.15 (-0.25, -0.04), *P *= 0.006, -0.24 (-0.38, -0.10), *P *= 0.001]. PSQI question 2 (sleep latency) was significantly lower across study visits in the PRM group in the age 65-80 population [-12.1 min (-19.1, -5.1), *P *= 0.001].

PRM patients aged 65-80 years were significantly more alert in the morning than placebo patients [-0.10 (-0.19, -0.01), *P *= 0.032; Table 6]. The effects of treatment on morning alertness were enhanced during the long term period as evidenced by a linear trend in treatment by visit effects (*P *= 0.012), with greater benefits for PRM patients at later visits. CGI-I scores were significantly lower (improved) across study visits in the PRM group compared with placebo for these patients [-0.20 (-0.38, -0.02), *P *= 0.027; Table 7].

### Safety

#### Treatment period

The safety population consisted of 789 patients who received at least one dose of study drug. A total of 31 patients reported 42 SAEs during the study, including one death in a placebo-treated patient. The incidence of non-fatal SAEs and discontinuations due to AE was low. One 68-year-old woman treated with PRM had an SAE of palpitations during the extension period of the study, which was assessed as possibly drug-related and was reported as a suspected unexpected serious adverse reaction (SUSAR). This patient had had a medical history of palpitations for 3 years prior to the study.

A total of 59 patients discontinued treatment with study drug due to an AE, 17 (9, 2% PRM; 8, 2% placebo) in the 3-week treatment period and 42 (30, 5.6% PRM; 12, 6.8% placebo) during the extension period. Overall, approximately 35% of patients had an AE during the 3-week treatment period; 75% during the 26-week extension period and 15% during the run-out period. AE rates were generally similar in the PRM and placebo treatment groups (Table 8).

**Table 8:** Number (%) of patients who had an adverse event (AE) in any category in the treatment and extension periods, safety population.

Category of AE	Treatment period		Extension period	
	
	PRM	Placebo	PRM	Placebo
No. of patients	394	395	534	177
Any AE	136 (34.5%)	142 (35.9%)	394 (73.8%)	136 (76.8%)
Any SAE	1 (0.3%)	3 (0.8%)	15 (2.8%)	9 (5.1%)
SAE leading to death	0 (0.0%)	0 (0.0%)	0 (0.0%)	1 (0.6%)
DAE	9 (2.3%)	8 (2.0%)	30 (5.6%)	12 (6.8%)
Drug-related AE*	17 (5.3%)	19 (6.1%)	56 (12.9%)	24 (17.3%)
Drug-related SAE*	0 (0.0%)	0 (0.0%)	1 (0.2%)	0 (0.0%)

Percentages based on number of patients in the safety population for each treatment group.

*Assessed by the investigator as definitely, probably or possibly related to study drug.

SAE, serious adverse event; DAE, premature discontinuation of treatment with investigational product due to an AE; PRM, prolonged release melatonin.

The number and percentage of patients with the most commonly reported system organ classes in the treatment and extension periods in the safety population are depicted in Table 9. Nasopharyngitis, arthralgia, diarrhoea, lower and upper respiratory tract infections and headache were the most commonly reported AEs in both the PRM and placebo-treated groups during both treatment (3-week and 26 weeks extension) periods (Table 9). There was no evidence of a difference between treatments or age groups in the type and amount of AEs.

**Table 9:** Number (%) of patients with the most commonly reported system organ classes in the treatment and extension periods, safety population.

System organ class	Treatment period		Extension period	
	
	PRM(*n *= 394)	Placebo(*n *= 395)	PRM(*n *= 534)	Placebo(*n *= 177)
Infections and infestations	38 (9.6)	43 (10.9)	176 (33.0)	60 (33.9)
Gastrointestinal disorders	18 (4.6)	31 (7.8)	113 (21.2)	37 (20.9)
Musculoskeletal and connective tissue disorders	21 (5.3)	21 (5.3)	113 (21.2)	34 (19.2)
Nervous system disorders	19 (4.8)	23 (5.8)	47 (8.8)	19 (10.7)
Respiratory, thoracic and mediastinal disorders	12 (3.0)	16 (4.1)	47 (8.8)	18 (10.2)
Skin and subcutaneous tissue disorders	12 (3.0)	9 (2.3)	43 (8.1)	13 (7.3)
Injury, poisoning and procedural complications	8 (2.0)	2 (0.5)	35 (6.6)	13 (7.3)
Renal and urinary disorders	9 (2.3)	4 (1.0)	29 (5.4)	7 (4.0)
General disorders and administration site disorders	5 (1.3)	5 (1.3)	28 (5.2)	16 (9.0)
Investigations	5 (1.3)	7 (1.8)	24 (4.5)	9 (5.1)
Eye disorders	5 (1.3)	1 (0.3)	20 (3.7)	6 (3.4)
Psychiatric disorders	5 (1.3)	4 (1.0)	17 (3.2)	13 (7.3)
Vascular disorders	2 (0.5)	0 (0)	16 (3.0)	5 (2.8)
Cardiac disorders	0 (0)	1 (0.3)	15 (2.8)	2 (1.1)
Ear and labyrinth disorders	0 (0)	1 (0.3)	13 (2.4)	2 (1.1)
Reproductive system and breast disorders	3 (0.8)	3 (0.8)	12 (2.2)	1 (0.6)
Metabolism and nutrition disorders	1 (0.3)	2 (0.5)	7 (1.3)	4 (2.3)
Surgical and medical procedures	1 (0.3)	2 (0.5)	7 (1.3)	6 (3.4)

PRM, prolonged release melatonin.

Changes in clinical laboratory results, including endocrine function (prolactin, ACTH, T3, free T4, TSH, LH, FSH), estradiol (women), free and total testosterone (men),and cortisol (before and after synacthen test), were generally small and showed no treatment-related trends. In particular, there were no apparent clinically relevant differences between the treatment groups in mean change from baseline for any endocrine function assessment for men aged > 50 and < 50 or post-menopause and pre-menopause women. In general, there were no apparent differences between treatment groups in vital signs, ECG, physical examination or any of the safety outcomes measured.

#### Withdrawal period

There was no evidence of a difference between treatment groups in the proportion of subjects experiencing new symptoms on the Tyrer questionnaire after the withdrawal period (secondary efficacy endpoint), which was about 28% in both groups (*P *= 0.881) indicating no withdrawal effects. However, placebo patients tended to exhibit more symptoms assessed by the Tyrer questionnaire at the end of both the extension period (treatment week 29; 49.2% reports on symptoms in the PRM versus 59.5% in the placebo group) and the end of the 2 weeks run-out period (withdrawal; 44.0% reports on symptoms in the PRM versus 50.4% in the placebo group). This was even more pronounced in the older population (aged 65-80) with 53.7% symptoms recorded in the PRM at the end of the extension period (treatment week 29) versus 67.7% in the placebo group and 45.4% symptoms recorded in the PRM versus 64.5% in the placebo group at the end of the 2 weeks run-out period (withdrawal). In this population the proportion of subjects experiencing new symptoms on the Tyrer questionnaire after the withdrawal period was 24.5% in the PRM group versus 35.5% in the placebo group.

## Discussion

To our knowledge, this is the first double-blind, placebo-controlled randomized trial evaluating the long-term effects of melatonin treatment in insomnia patients. A total of 722 of the 791 randomized patients were analysed in the full analysis set in this study with a DSM IV diagnosis of primary insomnia and a sleep latency of at least 20 min (mean sleep latency 74 min).

The main aim of this study, based on the shortening of sleep latency, was to determine if endogenous melatonin level regardless of age is useful to predict response to PRM therapy. The results provide evidence that short-term (3 weeks) treatment with PRM is effective in elderly patients. Notably, the age cutoff for patients >65 years used for the primary analysis in this study, does not preclude response to PRM in younger patients. Rather, there is sufficient evidence in previous studies [21-25] for an equal or greater response to PRM in patients aged 55 and older. Thus, further studies of the age cut-off for response are warranted.

The study also indicates that low melatonin excretion level regardless of age is not useful in predicting the response to PRM in insomnia. It is well known that melatonin levels vary widely between individuals [17,34]. There is also evidence that, regardless of initial levels at young age, there is a reduction in melatonin levels with age [11-18]. It has thus been proposed that a melatonin deficiency should be viewed as relative to melatonin levels at young age (a decline from 120 pg/mL when young to 40 pg/mL when old) rather than as absolute levels measured at old age [35]. The individual decline in melatonin production capability may better correspond to poor quality of sleep in insomnia patients than absolute melatonin levels [18,34]. Thus, the strong finding of age related efficacy may reflect within patient reduction in melatonin levels which is masked in the younger age groups by the wide variability in normal levels.

The effects of PRM on sleep latency in the 65-80-year-old patients are very similar to those found with current hypnotic drugs, including those developed primarily for patients with difficulty falling asleep [36-38]. The PRM efficacy reported in the present study is not only statistically significant but also clinically relevant. Furthermore, there were no signs of tolerance, as there was no reduction in benefit during the long-term treatment.

A limitation of this study was the lack of polysomnographic or actigraphic data. However, in clinical practice, patients with insomnia do not receive overnight sleep recordings and physicians base the success of any given treatment on patient reports of improved sleep and well being [39]. Furthermore actigraphy is considered less useful for sleep latency in insomnia [40]. Evidently the hypnotic effect of PRM in this study as measured by subjective means is very much like that documented in the sleep laboratory and previous studies using clinical assessments [24,25]. Therefore, the subjective improvements in this study are relevant and allow a better understanding the efficacy of PRM in the treatment of insomnia.

The magnitude of the effects of PRM on sleep latency documented in the present study, agree well with the results of the previous clinical trials with this drug [24], including a sleep laboratory study using polysomnography [25] in patients aged 55 and older. The observed effect on sleep latency for patients aged 65-80 years (-15.6 min; 95% CI -25.3 to -6.0, *P *= 0.002) was larger than would be predicted according to the published meta-analysis on efficacy of exogenous melatonin for primary insomnia [-7.2 min (95% CI -12.0, -2.4; *n *= 12)] [20] which was predominantly based on studies in younger patients and their use of immediate release preparations. The apparently weaker efficacy profile of 3 weeks treatment with PRM in the low excretors (18-80 years) may thus be due to the younger patients (aged <55 years) in the population.

There is evidence of improvements of additional sleep and daytime parameters with PRM. Global PSQI scores were improved in PRM patients aged 65 and older, in both the short and long term. Notably, in this trial, patients with poor quality of sleep alone were not entered; patients had also to have some difficulty in falling asleep to be included. It is therefore not surprising that in the short term, these overall effects may mainly be driven by reductions in sleep latency (component 2). In the long term, however, there was evidence of treatment benefits with respect to both sleep latency and sleep quality (component 1). There was also evidence of a delayed effect of PRM treatment in improve morning alertness (as measured by the diary) over the 6-month treatment period. Improvement in sleep quality and morning alertness were consistently found in clinical trials with PRM [23-25] but not in studies with immediate release melatonin formulations or the MT1/MT2 melatonin receptor agonist ramelteon [20,38,41]. Such improvements were also difficult to demonstrate with other insomnia drugs. Furthermore, the benefit of PRM in the patients' overall clinical status (as measured by the CGI-I scores) improved after long-term treatment, in patients aged 65-80 years.

Low excretors also had benefitted from PRM in the short and long periods. However, we concluded that the measurement of melatonin metabolite is not helpful in predicting response to PRM therapy.

A rather unexpected aspect of PRM efficacy is the incremental nature of the response with time. In addition, there seems to be a significant urge to advance bedtime with this treatment. These findings may indicate an effect on the treatment on the internal temporal order. There is a great deal of evidence indicating that aging is characterized by a progressive deterioration of circadian timekeeping, including loss of SCN melatonin receptors [14,42-44]. Functional disturbances of SCN circadian activity may start already around the age of 50, as evidenced by a decrease in vasopressin rhythmic function, in the expression of melatonin receptors in the SCN and in melatonin production compared to younger adults [14,16,43]. Consequently, there is disorganization of the internal temporal order [12,44]. PRM treatment has been shown to delay the nocturnal cortisol production in elderly insomnia patients towards the morning [45], improve blood pressure rhythms [46] and, as shown here, progressively advance time to bed in those aged 65-80 years old in addition to the shortening of sleep latency. Improvement in internal temporal order with PRM may explain why the treatment effects are more prominent in older patients and there is a gradual development of response over days or weeks [25]. Further research will aim at investigating whether the evolution of response to PRM represents a re-activation of the circadian system or reinforcement of responsiveness to melatonin.

There were no new or unexpected safety findings following short- and long-term treatment with PRM, except for one case of palpitations. The safety profile of the PRM treatment group was very similar to placebo with respect to the incidence and type of AEs, the results of clinical laboratory tests, vital signs, ECG and physical examination and endocrine function including prolactin, ACTH, T3, free T4, TSH, LH, FSH, oestradiol (women), free and total testosterone (men) and cortisol (before and after synacthen test). There were no withdrawal effects after ceasing long-term treatment with PRM, as assessed by the Tyrer questionnaire scores or incidences of AEs during the run-out period. The improvements in sleep parameters decreased during the run-out period but had not reached baseline values by the end of this period.

## Conclusions

Patients aged 65 years and over with primary insomnia are likely to have a good response to melatonin therapy and the response will increase and be sustained over a period of 6 months. Further studies of the age cut-off for response are warranted. A low melatonin excretion level, regardless of age, is not useful in predicting response to PRM in insomnia and this is probably due to wide variation among individuals. Thus, clinicians do not require melatonin measurements prior to treatment. There were no rebound or withdrawal effects upon the discontinuation of PRM following long-term use.

The safety and efficacy profile of PR melatonin, as demonstrated in this study, supports its continuous use for 6 months in the treatment of primary insomnia in the target population.

## Abbreviations

6-SMT: 6-sulphatoxymelatonin; AE: adverse effect; CGI-I: Clinical Global Impression of Improvement; CGI-S: Clinical Global Impression of Severity of Illness Scale; DSM: Diagnostic and Statistical Manual for Mental Disorders; FAS: Full Analysis set; IVRS: Interactive Voice Response System; MMRM: mixed effects model for repeated measures; PRM: prolonged release melatonin; PSQI: Pittsburgh Sleep Quality Index; SAE: serious AE; SCN: suprachiasmatic nuclei; SHQ: Sleep History Questionnaire.

## Competing interests

All authors declare that they have disclosed all their competing interests. Financial support for the submitted work was provided by Neurim Pharmaceuticals. AGW and IF have acted as paid consultants to Neurim Pharmaceuticals. AGW and GC are owners of the company which was responsible for carrying out the clinical study. TN and ML are employees of Neurim Pharmaceuticals. NZ is the founder and Chief Scientific Officer of Neurim Pharmaceuticals. AM declares no interests and all authors (except NZ) declare that they have no spouses, partners or children with relationships with commercial entities that might have an interest in the submitted work.

## Authors' contributions

AGW was responsible for the integrity of the work as a whole, from inception to the published article. AGW, IF, NZ, TN and ML made substantial contributions to conception and design of the study and were involved in drafting the protocol, the interpretation of data and the preparation of the manuscript. AGW and GC were responsible for the acquisition of data and patient care. IF and AM were responsible for the statistical plan, blinded data archiving and all statistical analyses, revised the manuscript critically for important intellectual content and had access to the data. All authors approved the final version. All authors had full access to all of the data post un-blinding (including statistical reports and tables) in the study and can take responsibility for the integrity of the data and the accuracy of the data analysis

## Pre-publication history

The pre-publication history for this paper can be accessed here:

http://www.biomedcentral.com/1741-7015/8/51/prepub
